# Focused Ultrasound in Pancreatic Ductal Adenocarcinoma: Mechanisms, Preclinical Evidence, and Emerging Clinical Applications

**DOI:** 10.3390/cancers18040574

**Published:** 2026-02-10

**Authors:** Olivia Sears, Hongji Zhang, Natalie Blatz, Xiao Cui, Allan Tsung

**Affiliations:** Department of Surgery, University of Virginia, Charlottesville, VA 22904, USA; jhn5wx@virginia.edu (H.Z.); nb7eut@uvahealth.org (N.B.); hwy4sp@virginia.edu (X.C.); crf9aa@uvahealth.org (A.T.)

**Keywords:** pancreatic ductal adenocarcinoma, focused ultrasound, high-intensity focused ultrasound, histotripsy, tumor microenvironment, drug delivery, immunotherapy, image-guided ablation, sonoporation

## Abstract

Pancreatic ductal adenocarcinoma (PDAC) remains a highly lethal malignancy due to late presentation, limited resectability, therapeutic resistance, and a dense desmoplastic immunosuppressive tumor microenvironment that impairs drug penetration and antitumor immunity. Focused ultrasound (FUS) is an emerging non-invasive, image-guided therapeutic platform capable of delivering spatially confined acoustic energy to induce tumor ablation, disrupt stromal barriers, and enhance delivery of drugs, nanoparticles, and nucleic acids. Depending on acoustic parameters, FUS can produce thermal effects resulting in coagulative necrosis or non-thermal mechanical effects, including cavitation, sonoporation, and histotripsy which remodel extracellular matrix architecture, increase vascular and cellular permeability, and facilitate tumor debulking. In addition, FUS-induced cell injury can promote immunogenic cell death and release tumor-associated antigens and danger signals, providing a rationale for combination strategies with chemotherapy, radiation, and immunotherapy. This review synthesizes the mechanistic foundations, preclinical modeling advances, and emerging clinical applications of FUS in PDAC, with emphasis on treatment integration, patient selection, real-time monitoring, and acoustic parameter optimization, while acknowledging current safety considerations and limited clinical toxicity data. Key limitations, translational challenges, and priority knowledge gaps are also discussed to define the role of FUS in multimodal PDAC care.

## Simple Summary

Pancreatic ductal adenocarcinoma is one of the deadliest cancers, in part because its dense structure limits the effectiveness of chemotherapy, radiation, and immunotherapy. Focused ultrasound is a non-invasive technology that uses sound waves to precisely target tumors without surgery. Depending on how it is applied, focused ultrasound can destroy tumor tissue, disrupt physical barriers that block drug delivery, and stimulate immune responses against cancer. In recent years, focused ultrasound has shown promise in laboratory models and early clinical studies for pancreatic cancer. This review summarizes how focused ultrasound works, what has been learned from preclinical and clinical studies, and where the field is headed. Understanding how this technology can be integrated into pancreatic cancer care may help expand treatment options for patients with otherwise limited therapeutic choices.

## 1. Introduction

Pancreatic ductal adenocarcinoma (PDAC) remains one of the most lethal malignancies, with a 5-year overall survival rate between 10 and 13% and limited therapeutic progress over the past several decades [1,2]. Over 80% of patients are diagnosed with either locally advanced or metastatic disease at presentation. This renders surgical resection, the only curative option, nonviable in the majority of cases [1,3]. Standard chemotherapeutic regimens including FOLFIRINOX or gemcitabine/nab-paclitaxel have demonstrated modest improvements in survival but are often constrained by systemic toxicity and therapeutic resistance [2].

The desmoplastic and immunosuppressive tumor microenvironment (TME) in PDAC is a major contributor to therapeutic failure. Activated stellate cells and fibrotic stroma create a hypovascular, immunosuppressive microenvironment that hinders drug penetration and promotes tumor aggressiveness [4,5]. These features not only compromise the efficacy of chemotherapy and immunotherapy but also limit radiation dosing due to the proximity of critical structures in the retroperitoneum [5]. As a result, local disease progression and treatment resistance remain dominant clinical challenges even in the era of multi-agent systemic therapy.

Locoregional therapies including irreversible electroporation (IRE), radiofrequency and microwave ablation (RFA/MWA), and stereotactic body radiation therapy (SBRT) have been explored in PDAC with mixed results [5]. IRE can achieve local control but is technically demanding and limited by incomplete ablation in fibrotic tissue [6]. RFA/MWA are restricted by heat-sink effects near major vessels, and SBRT is constrained by dose-limiting exposure of adjacent gastrointestinal organs [5,7]. Collectively, these limitations underscore the need for non-invasive, image-guided approaches capable of penetrating stromal barriers while minimizing off-target injury.

**Figure 1:**
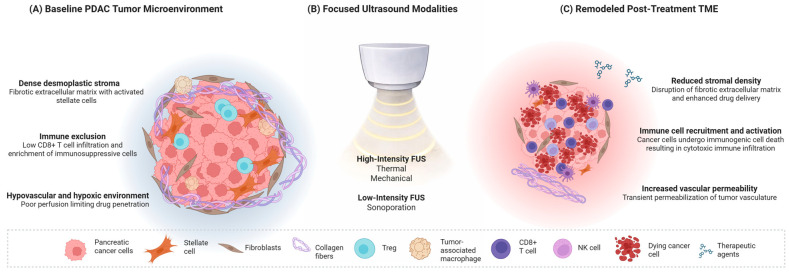
Focused ultrasound (FUS) as a multimodal platform for remodeling the pancreatic ductal adenocarcinoma (PDAC) tumor microenvironment (TME). (**A**) PDAC is characterized by dense desmoplastic stroma, hypovascularity, and immune exclusion which limit drug delivery and immunotherapy efficacy. (**B**) FUS can be delivered using distinct acoustic regimens, including thermal ablation, mechanical disruption, and sonoporation. (**C**) These modalities promote cytoreduction, stromal remodeling, increased vascular and cellular permeability, enhanced intratumoral drug delivery, and immune activation, therefore supporting integration of FUS into multimodal treatment strategies for PDAC.

Among emerging technologies, focused ultrasound (FUS) has gained momentum as a non-invasive, image-guided platform capable of delivering spatially and temporally controlled acoustic energy to deep tissues. Depending on acoustic parameters, FUS can induce thermal ablation, non-thermal mechanical disruption, or transient increases in vascular and cellular permeability, thereby enabling tumor cytoreduction, stromal remodeling, and enhanced delivery of therapeutic agents (Figure 1). Real-time guidance using ultrasound (US), magnetic resonance imaging (MRI), or endoscopic ultrasound (EUS) allows precise targeting while sparing surrounding structures [8,9].

FUS can generate therapeutic effects via both thermal and mechanical mechanisms. Thermal ablation induces coagulative necrosis and has shown synergy with chemotherapy in preclinical and early clinical studies [10,11]. Mechanical mechanisms, including cavitation and boiling histotripsy promote stromal remodeling, vascular permeabilization, and tumor debulking, while also facilitating enhanced delivery of therapeutics [12,13,14]. Importantly, FUS-induced cell lysis has been shown to release tumor-associated antigens (TAAs) and damage-associated molecular patterns (DAMPs), potentially enhancing immune recognition and sensitizing tumors to immunotherapy [15,16].

Together, these capabilities position FUS as a multifunctional platform well-suited to overcome PDAC’s dominant resistance mechanisms including stromal exclusion, impaired drug penetration and immune suppression. It achieves this through coordinated tumor ablation, enhanced drug delivery and immune priming. However, the biological effects, optimal treatment parameters, and clinical integration of FUS in pancreatic cancer remain incompletely defined.

In this review, we synthesize current knowledge on the mechanistic basis of FUS in PDAC, summarize advances in preclinical modeling, and critically examine emerging clinical applications. We place particular emphasis on integration with systemic therapies, imaging and acoustic parameter optimization, patient selection, and translational barriers. Key limitations, regulatory considerations, and priority knowledge gaps are highlighted to inform future research and define the potential role of FUS within multimodal PDAC management.

## 2. Mechanistic Basis of Focused Ultrasound in PDAC

Throughout this review, focused ultrasound (FUS) is used as an umbrella term encompassing all ultrasound-based therapeutic strategies. High-intensity focused ultrasound (HIFU) refers specifically to thermal ablation using continuous or high-duty cycle exposure. Pulsed high-intensity focused ultrasound (pHIFU) describes low-duty cycle exposures that exert mechanical rather than thermal effects, such as stromal disruption. Low-intensity focused ultrasound (LIFU) and sonoporation are used to describe reversible membrane permeabilization techniques, typically requiring contrast agents like microbubbles. Histotripsy represents a distinct, non-thermal, non-invasive method of mechanical tissue fractionation using ultrashort, high-pressure pulses. A comparative summary of mechanistic distinctions, clinical goals, and current limitations of each FUS modality in PDAC is provided in Table 1 and expanded upon in this section.

### 2.1. Thermal Ablation

Thermal ablation via FUS delivers concentrated acoustic energy to a targeted region, inducing localized hyperthermia. This results in coagulative necrosis of tumor tissue while generally preserving adjacent structures. Temperatures typically exceed 55 °C, creating well-defined ablation zones with clear margins and minimal injury to surrounding tissue [17]. The ability to focus ultrasonic energy allows rapid heating within the focal zone while sparing overlying and adjacent tissues through a steep thermal gradient and real-time image guidance [18].

In PDAC, thermal ablation has shown the potential to reduce tumor volume, relieve pain, and improve short-term outcomes, particularly in unresectable cases [18,19,20]. Early clinical applications of HIFU have demonstrated its feasibility for thermal ablation in PDAC. Ran et al. reported that combining HIFU with pharmacogenomic-guided chemotherapy improved local control and was well-tolerated in advanced-stage patients [11]. Similarly, Zhou et al. described a multicenter consensus on US-guided HIFU, emphasizing its utility in reducing tumor burden and improving drug sensitivity when used adjunctively with systemic therapy [18]. A meta-analysis of HIFU for locally advanced PDAC also reported consistent improvements in pain control and local tumor response with low complication rates, further supporting the safety and feasibility of thermal ablation in this context [21].

Beyond cytoreduction, thermal ablation may modulate the tumor microenvironment (TME). By inducing vascular disruption, hyperthermia can increase tumor perfusion and facilitate chemotherapeutic delivery [5,22]. Additionally, thermal injury can trigger immunogenic cell death (ICD), promoting antigen release and dendritic cell activation [23]. These effects suggest a synergistic role for thermal FUS in combination protocols with chemotherapy and immunotherapy.

However, thermal ablation in PDAC faces anatomical constraints. The retroperitoneal location of the pancreas, proximity to gastrointestinal structures, and high stromal content can lead to energy attenuation and incomplete ablation zones. Human pancreatic tissue exhibits lower sound speed and higher attenuation, previously reported in non-human studies, with simulations showing approximately 40% smaller ablation volumes than predicted in preclinical data [24]. Careful acoustic parameter selection and real-time monitoring remain critical for maximizing efficacy while minimizing off-target injury.

### 2.2. Mechanical Disruption

Mechanical effects of FUS occur when acoustic energy is delivered at lower-duty cycles and intensities that do not produce sustained heating. Instead, rapid pressure fluctuations induce non-thermal mechanisms such as cavitation, acoustic streaming, and mechanical shearing, resulting in direct tumor disruption and stromal remodeling [9,22,25].

Cavitation is central to mechanical ablation. Inertial cavitation generates microbubble collapse and shock waves that fragment tissue and lyse tumor cells, and enhance the permeability of the extracellular matrix [26,27]. Boiling histotripsy uses high-pressure, short-pulse energy to create vapor bubbles that mechanically fractionate tissue without significant temperature elevation [28]. These approaches offer non-thermal alternatives to conventional ablation, particularly in highly fibrotic tumors.

In PDAC, mechanical FUS has demonstrated the ability to disrupt the dense stroma that hinders drug and immune cell access. In orthotopic murine models, microbubble-enhanced sonoporation improved chemotherapy penetration and reduced hypoxia within the tumor [13]. Pulsed HIFU treatment associated with high cavitation activity resulted in disruption of the highly fibrotic stromal matrix and was shown to enhance doxorubicin concentrations by up to 4.5-fold compared with controls [27]. Parameter optimization has also been shown to increase cellular permeability without causing collateral tissue damage [29].

Larger tumor debulking is feasible using mechanical approaches. Preclinical studies in porcine models confirmed that histotripsy can ablate pancreatic tissue with high precision and minimal damage to surrounding structures [14,28]. Unlike thermal methods, mechanical disruption is less influenced by tissue perfusion and can produce more uniform treatment effects in tumors with variable stromal architecture [24].

Challenges remain especially in maintaining safe cavitation thresholds and ensuring adequate visualization. Emerging real-time monitoring tools, such as passive cavitation detectors and contrast-enhanced ultrasound imaging, are being explored to enhance safety and control [9,15,30,31].

### 2.3. Enhanced Drug and Gene Delivery

FUS can enhance the delivery of therapeutic agents into pancreatic tumors by transiently increasing vascular and cellular permeability. This is primarily achieved through low-intensity focused ultrasound (LIFU) mechanisms such as sonoporation, a process in which acoustic pressure induces the formation of transient pores in cell membranes and vasculature. This allows for improved extravasation and intracellular uptake of systemically administered drugs without causing irreversible tissue injury [15,27,32].

Microbubble-mediated sonoporation has been extensively studied in preclinical PDAC models. Li et al. demonstrated that pulsed HIFU improved intratumoral accumulation of doxorubicin, resulting in increased cytotoxicity and extended survival in murine xenografts [27]. Similarly, Park et al. reported that US-guided FUS enhanced gemcitabine delivery and therapeutic response in orthotopic models [33]. More recently, Doppler-based LIFU exposure in combination with microbubbles has been shown to relieve hypoxia and improve the efficacy of chemotherapy and immunotherapy in murine PDAC models [13]. Collectively, these studies support a consistent role for FUS in overcoming delivery barriers imposed by the dense pancreatic stroma.

Beyond free drug delivery, FUS has been used to facilitate the targeted release of nanoparticle and liposomal formulations. These carriers can be engineered for stability in circulation and triggered for release upon exposure to ultrasound energy. Fang et al. developed ultrasound-responsive lipid nanosensitizers that enabled on-demand delivery of nitric oxide and chemotherapeutic payloads, enhancing sonodynamic and immune responses in pancreatic tumors [34]. Conte et al. reported that acoustically activated hybrid nanocrystals achieved deep tumor penetration and effective drug delivery in vivo [35]. These approaches rely on the low acoustic pressure of pulsed LIFU to selectively trigger drug release from thermosensitive or acoustically sensitive constructs, enabling localized therapy with minimal off-target effects.

FUS has also been applied to enhance gene transfection in pancreatic cancer. Although still in early development, preliminary studies indicate that FUS can facilitate the intracellular uptake of plasmids and small interfering RNAs through mechanical disruption of cell membranes. LIFU offers particular promise in this domain by promoting transfection with lower cytotoxicity compared to electroporation or viral vectors, with the potential for repeated dosing if necessary [36].

Limitations include the need for precise control of acoustic parameters to avoid vascular damage or off-target effects. The size, composition, and stability of drug carriers also influence delivery efficiency. Real-time imaging and acoustic monitoring are essential for optimizing these parameters and ensuring safe, localized delivery.

### 2.4. Acoustic Exposure Strategies Across Applications

The acoustic parameters used in FUS vary significantly depending on the intended mechanism of action and clinical target. In PDAC, these parameters are carefully modulated due to the pancreas’s anatomic proximity to gastrointestinal and vascular structures and its high acoustic attenuation. Treatment duration varies substantially by FUS modality and delivery platform, ranging from seconds to minutes for mechanical approaches to several hours for thermal ablation regimens. A representative comparison of parameter ranges is presented in Table 2.

Thermal ablation typically employs spatial peak pulse average intensities (ISPPA) in the range of 1.5–2.5 kW/cm^2^ in PDAC, which are lower than in other abdominal tumors. Intermediate ISPPA values (e.g., 2.0 kW/cm^2^) with low-duty cycles (approximately 1%) have demonstrated favorable results [24,37]. This includes >30% tumor volume reduction and survival benefit in early-phase clinical trials. Several HIFU systems developed in China deliver average powers of 225–260 W and total energies of 70–1195 kJ across multiple hours of treatment [19,38].

Mechanical disruption, particularly using pulsed HIFU, uses negative pressures around 16.5 MPa, 1 ms pulses, and 1% duty cycles. This approach enhances drug delivery by disrupting the dense stromal matrix of PDAC, increasing the intratumoral penetration of chemotherapeutics such as gemcitabine or doxorubicin by up to 4.5-fold [27,39]. Lower mechanical intensities (e.g., 2.0 kW/cm^2^ ISPPA) also show enhanced drug uptake of about 1.5-fold with improved safety relative to thermal regimens [29].

Sonoporation techniques span both extracorporeal and endoscopic modalities. Extracorporeal sonoporation uses acoustic intensities between 60 and 200 mW/cm^2^ with frequencies of 1–3 MHz. Higher output (200 mW/cm^2^) has been more consistently associated with tumor suppression [40,41]. EUS-guided approaches allow for greater spatial precision, generating peak negative pressures up to 6.55 MPa at short focal distances (approximately 20 mm) [30].

Histotripsy employs microsecond-length ultrasound pulses at approximately 500 Hz frequency, producing peak negative pressures > 15 MPa and pulse repetition rates of 100–500 Hz. These settings result in sharply demarcated spherical lesions (1.4–1.5 cm) visible on computed tomography (CT) in a porcine model [28]. The major challenge is ultrasound visualization with overlying bowel gas potentially causing off-target effects, suggesting mechanical bowel preparation will be valuable as this technology moves forward in clinical stages [14].

Finally, recent studies have highlighted that the acoustic properties of human pancreatic tissue, including sound speed and attenuation, differ from animal models. This discrepancy leads to an approximate 30% reduction in thermal effect transmission in clinical settings but exerts minimal influence on cavitation thresholds relevant for histotripsy [24].

### 2.5. Immune Modulation

FUS has demonstrated the capacity to modulate the tumor immune microenvironment through both direct and indirect mechanisms. Acoustic energy can trigger immunogenic cell death [ICD], promote antigen release, and increase trafficking of immune cells into previously immune-poor tumors.

Thermal and mechanical FUS exposures have been shown to release tumor-associated antigens (TAAs) and damage-associated molecular patterns (DAMPs), including HMGB1, calreticulin, and ATP. These signals facilitate dendritic cell maturation and antigen presentation, enhancing T cell priming in preclinical models of PDAC and other solid tumors [12]. Mouratidis et al. observed that FUS treatment in orthotopic PDAC increased intratumoral CD8+ T cells and reduced Treg population, particularly when combined with anti-programmed cell death protein-1 (PD-1) therapy [42].

Histotripsy in particular has emerged as a potent immune stimulant. In murine melanoma and hepatocellular carcinoma models, histotripsy-induced tumor ablation led to increased expression of pro-inflammatory cytokines, dendritic cell activation, and infiltration of cytotoxic lymphocytes [12,14]. While data in PDAC are limited, similar immune-activating effects have been reported following mechanical FUS in preclinical pancreatic models [42].

The potential for FUS to induce systemic immune responses has been highlighted by reports of abscopal effects in nonpancreatic tumor models. In bilateral breast cancer models, combination FUS and checkpoint blockade produced tumor regression at both treated and untreated sites, suggesting enhanced systemic antigen presentation and T cell trafficking [43]. While abscopal effects remain rare in PDAC due to its low neoantigen burden and suppressive stroma, FUS-mediated priming could improve responsiveness to immunotherapy if paired with checkpoint inhibitors or oncolytic viruses.

Challenges include variability in the timing and consistency of immune activation, incomplete ablation in fibrotic tumors, and the need for appropriate sequencing with immunotherapeutic agents. Despite these limitations, FUS offers a non-invasive, spatially targeted strategy to reprogram the PDAC immune environment with ongoing trials investigating its role in combination immunotherapy [34,44].

While preclinical studies strongly support the diverse capabilities of FUS, many of these data are limited to rodent and porcine models. The clinical relevance of these mechanisms remains speculative without large-animal or early-phase human validation. Furthermore, FUS modalities have not been compared head-to-head in PDAC models, limiting the ability to tailor technique selection to tumor biology. Translation from these mechanistic insights into standardized protocols will be an important objective in the field.

## 3. Preclinical Evidence in PDAC Models

Robust preclinical models are essential for evaluating the biological effects, safety, and translational potential of FUS in pancreatic cancer. Murine models remain the most widely used due to cost-effectiveness, genetic flexibility, and compatibility with immunological studies. Orthotopic xenografts in particular replicate the anatomical constraints, desmoplastic stroma, and vascular characteristics of human PDAC compared to subcutaneous implants, providing a more clinically relevant platform for assessing FUS treatment [45].

In immunocompetent murine models, FUS has been used to evaluate chemotherapy delivery, stromal modulation, and immune activation. Wu et al. demonstrated enhanced doxorubicin accumulation and reduced hypoxia following microbubble-assisted sonoporation in orthotopic PDAC tumors, with increased survival compared to controls [13]. Other studies using syngeneic models have shown FUS-induced T cell infiltration and improved response to checkpoint inhibitors [16,42]. These models have also enabled exploration of sonodynamic therapy, where FUS triggers cytotoxic activity of sensitizers localized in the tumor [44].

The continued development of genetically engineered mouse models (GEMMs), particularly those recapitulating KRAS and TP53 mutations, will be critical for future studies evaluating FUS in combination with targeted and immunotherapies. Such models offer the opportunity to assess long-term tumor evolution, treatment resistance, and immune memory in a physiologically relevant context [24].

Despite their utility, small-animal models are limited by anatomical differences, particularly in tissue depth, organ motion, and scale of energy deposition. To address this, large-animal models such as pigs have been employed to validate FUS delivery systems, assess safety margins, and simulate clinically relevant treatment conditions. Marinova et al. demonstrated the feasibility of EUS-guided HIFU (EUS-FUS) in a porcine model, achieving controlled ablation zones with minimal off-target effects. Others have used porcine pancreas models to validate acoustic parameter optimization and target accuracy for histotripsy [14,28,30].

Advanced imaging techniques, including MRI thermometry and contrast-enhanced ultrasound, have been incorporated into preclinical workflows to enable real-time monitoring of treatment efficacy and cavitation dynamics [9,40]. These tools improve reproducibility and allow correlation of imaging biomarkers with histopathologic and immunologic endpoints.

Together, the preclinical landscape has demonstrated that FUS can modify stromal structure, enhance drug accumulation, stimulate antitumor immunity, and achieve precise ablation. These findings provide mechanistic foundation for ongoing clinical translation.

## 4. Clinical Applications

### 4.1. Pain Palliation and Symptom Management

Clinical adoption of FUS in PDAC remains in the early stages but has shown feasibility, safety, and promising adjunctive benefit in several studies (Table 3). Most clinical applications to date have focused on HIFU for tumor ablation and pain palliation in patients with unresectable or locally advanced disease [18,21].

The largest published experience comes from international cohorts, with particularly high case volumes reported in East Asia, where US-guided HIFU is more widely integrated into clinical practice. A multicenter retrospective study of 523 patients reported improved pain control in 80% of cases and median survival of 12.2 months, with minimal major complications. Adverse events included transient abdominal pain, low-grade skin burns, mild pancreatitis, and reported rates of grade ≥ 3 adverse events were below 5% [46].

HIFU has shown utility for symptom palliation in patients with malignant biliary obstruction due to cholangiocarcinoma. Recent prospective trials and meta-analyses demonstrate that USgHIFU, when combined with biliary stenting, can relieve biliary obstruction and improve quality of life in patients with unresectable disease by reducing tumor burden along the biliary tree. This approach has been shown to prolong stent patency and overall survival compared to stenting alone, without increasing complication rates [47,48]. These findings are particularly relevant to PDAC, where malignant biliary and duodenal obstruction frequently impact quality of life. Adaptation of HIFU for biliary decompression or stent patency in PDAC could offer a non-invasive alternative to repeated interventions or surgical bypass, especially in poor surgical candidates.

### 4.2. Combination with Systemic Therapies

FUS has also been investigated in combination with chemotherapy in PDAC. In a prospective study by Ran et al., HIFU combined with chemotherapy based on pharmacogenomic profiling significantly improved disease control rates compared to chemotherapy alone [68% versus 45%] in patients with locally advanced PDAC [11]. Similarly, Marinova et al. reported improved local tumor response and acceptable safety following MRgHIFU plus gemcitabine in a Phase I cohort [20]. Early combination data support the premise that FUS may enhance drug delivery and improve local disease control when integrated into systemic therapy regimens.

At the time of this review, ongoing clinical trials are investigating FUS as a component of multimodal therapy. These include combinations with chemoradiation, immune checkpoint inhibitors, and sonodynamic therapy, which uses agents like indocyanine green or porphyrin derivatives to create ultrasound-activated reactive oxygen species (ROS). Early phase studies in hepatocellular carcinoma and breast cancer have suggested that FUS may augment systemic immune responses, and similar trial designs are now being applied to PDAC. Feasibility studies for histotripsy in the pancreas are underway, although clinical translation remains limited by anatomical constraints and regulatory clearance.

### 4.3. EUS-Guided Approaches

EUS-FUS is an emerging approach that allows for precise targeting of deep pancreatic lesions with improved safety profiles. Preclinical validation in porcine models has led to early human applications, with studies showing successful ablation zones and no adjacent organ injury [8,30]. EUS-based delivery may help overcome limitations in transabdominal acoustic access, particularly in patients with unfavorable anatomy or intervening bowel gas. These systems also create opportunities for integrated biopsy, targeted drug injection, or immunotherapeutic delivery during FUS exposure.

### 4.4. Comparative Perspective on Locoregional Therapies

FUS, irreversible electroporation (IRE), radiofrequency ablation (RFA), and stereotactic body radiation therapy (SBRT) represent distinct locoregional strategies for patients with locally advanced PDAC who are not surgical candidates. Among these modalities, SBRT currently has the strongest evidence of support and is incorporated into international treatment guidelines, with reported median overall survival (OS) ranging from 16 to 24 months and generally acceptable toxicity in appropriately selected patients [49].

IRE has demonstrated heterogenous survival outcomes and is associated with higher complication rates, reflecting both technical complexity and proximity to critical vascular structures. However, it may offer select benefit in perivascular tumors and is being actively explored in combination with immunotherapy [50]. RFA remains the least supported modality in PDAC, with inconsistent oncologic benefit and a comparatively unfavorable safety profile, limiting its adoption [51].

HIFU, delivered in combination with systemic chemotherapy, has shown encouraging outcomes in early clinical series, with reported median OS of 17–21 months and low rates of high-grade adverse events (<10%). However, these data derive predominantly from non-randomized cohorts and lack large-scale global validation [21]. Unlike IRE and RFA, FUS is non-invasive and better tolerated, but prolonged treatment times and acoustic access may limit candidate eligibility. While head-to-head data is lacking, ongoing trials may help clarify the optimal positioning of FUS relative to these locoregional therapies.

### 4.5. Global Experience and Limitations

Real-world data on FUS in PDAC remains sparse and geographically concentrated. Most published trials originate from East Asian centers, particularly China, where dedicated FUS systems and procedural experience have become more widely available. Western studies remain limited, and randomized controlled trials with long-term follow-up are lacking. As a result, the generalizability of current findings to broader healthcare systems and patient populations is uncertain. Further international trials will be essential to define standard of care applications.

## 5. Patient Selection and Clinical Integration

Focused ultrasound is not yet part of the standard PDAC treatment algorithms, but emerging evidence supports its potential role as a non-invasive adjunct. Appropriate integration requires consideration of anatomic, technical, and disease-specific factors.

### 5.1. Patient Selection

The selection of patients for FUS treatments in PDAC remains an evolving process, as no standardized criteria currently define a safe acoustic window or threshold distances from critical structures such as the duodenum, bowel, or major vessels. Most available guidance is extrapolated from early-phase studies, expert consensus, and institutional experience rather than formalized guidelines. While emerging data support the feasibility of treating tumors adjacent to vascular structures, safe patient selection remains highly dependent on individualized anatomic assessment and procedural risk mitigation. Defining and validating these thresholds through prospective studies will be essential to standardize patient eligibility and treatment safety moving forward.

Transabdominal HIFU is most feasible in patients with a clear acoustic window, stable respiratory motion, and minimal overlying bowel. Adequate acoustic window visualization is the primary imaging requirement for FUS in PDAC, with approximately 8–15% of patients in one study being excluded due to limited device accessibility from overlying bowel gas or anatomical constraints [52]. This requirement can be optimized through dietary preparation with simethicone and bisacodyl, as well as the use of hydrogel pads to displace the bowel and controlled respiration during the procedure [53]. EUS-guided systems may expand eligibility by enabling direct visualization and energy delivery to deep or obstructed regions of the pancreas [30,54].

Although no absolute minimum distance from major vessels is established, HIFU has been performed safely in select tumors abutting or encasing peripancreatic vessels. In one study, 13 of 15 patients had significant vessel involvement without hemodynamic changes, vascular stenosis, or adverse vascular events post HIFU [55]. Preclinical studies have been more conservative, demonstrating that higher energy doses (>30 kJ) near mesenteric vessels can induce transient arterial spasm. However, at lower settings, no vascular complications were observed [56]. These findings suggest that while vascular proximity is not an absolute contraindication, careful energy titration and real-time monitoring are essential to avoid complications.

### 5.2. Integration into Clinical Practice

Most clinical experience to date involves patients with locally advanced or unresectable PDAC who are not candidates for surgery but have preserved performance status. Potential indications include locally advanced tumors requiring cytoreduction to relieve pain or reduce local mass effect, enhancement of chemotherapy delivery in patients receiving neoadjuvant or palliative systemic therapy, adjunctive therapy in patients with persistent pain refractory to opioids or celiac plexus neurolysis and select cases of malignant biliary obstruction where FUS may prolong stent patency or reduce tumor ingrowth. Patients with extensive metastatic disease, poor acoustic windows, uncontrolled gastrointestinal obstruction, or poor performance status may be less suitable candidates.

FUS may complement existing treatment pathways in several settings. In the neoadjuvant setting, FUS may enhance intratumoral drug penetration and modulate the TME, potentially improving response to FOLFIRINOX or gemcitabine-based regimens. Early combination studies suggest higher disease control rates when FUS is added during treatment cycles [21,57]. For patients with locally advanced disease who are not candidates for surgery or for thermal ablation techniques, including IRE or RFA, FUS offers a non-invasive option for cytoreduction, pain relief, and local disease control particularly when anatomy may limit other modalities. Thermal HIFU has also demonstrated meaningful benefit for pain palliation and may reduce opioid requirements [46]. As discussed in Section 4.1, FUS has also been explored as an adjunct to biliary stenting in malignant obstruction, with early studies suggesting potential benefits in symptom control and stent patency [48,58,59]. Although still investigational, FUS is being evaluated in combination with immunotherapy as it may enhance checkpoint inhibitor responsiveness in an otherwise immunologically cold TME.

## 6. Technological Innovations and Emerging Systems

Recent technological advances have expanded the therapeutic precision and versatility of FUS platforms for PDAC. Developments in image-guided systems, nanotechnology, and energy modulation are enabling improved safety, efficacy and more accessible integration with multimodal therapies.

Image guidance is central to the clinical application of FUS in PDAC. While ultrasound-guided HIFU remains the most widely used in clinical cohorts, MRI-guided FUS (MRIgFUS) offers superior soft-tissue contrast and thermometry, allowing for accurate thermal dose monitoring and spatial control [9]. EUS-FUS has emerged as a promising platform for accessing deep retroperitoneal lesions with high targeting accuracy and reduced off-target injury. Early porcine studies and feasibility trials have shown EUS-FUS can safely deliver ablation to pancreatic lesions with minimal collateral damage, expanding eligibility to patients with poor transabdominal acoustic access [8,14,28,30].

Nanotechnology has broadened the scope of FUS beyond ablation by helping to improve drug delivery, imaging, and immune stimulation. Liposomal, polymeric, and lipid-based nanoparticles can be loaded with chemotherapeutics, immunostimulants, or sonosensitizers which are then triggered to release their payload upon acoustic activation. Fang et al. developed ultrasound-responsive lipid nanosensitizers for nitric oxide and drug delivery, while Conte et al. reported hybrid nanocrystals capable of deep stromal penetration and enhanced tumor uptake [34,35]. These constructs allow spatially controlled drug release, which may reduce systemic toxicity and improve treatment consistency across heterogeneous tissue.

Acoustic parameter optimization continues to evolve as a key area of translational research. Techniques such as passive cavitation imaging, real-time elastography, and adaptive feedback loops are being developed to refine energy delivery based on tissue characteristics. This is particularly important in PDAC, where dense stroma and variable perfusion can affect energy absorption and treatment consistency.

Collectively, these innovations are transforming FUS into a precision therapy platform capable of tailored ablation, targeted delivery, and real-time control. Continued refinement will be critical for clinical scalability and integration with systemic and immune-based treatment regimens.

## 7. Focused Ultrasound as an Immunotherapy Adjunct

Building upon the immune-activating mechanisms described in Section 2.5, FUS has emerged as a promising strategy to enhance immunotherapy responsiveness in PDAC. Checkpoint blockade and other immune-based therapies have shown limited efficacy due to low tumor immunogenicity and dense, suppressive stroma

Through leveraging previously demonstrated immune-priming effects, FUS is being evaluated as a strategy to enhance responsiveness to checkpoint blockade. In preclinical PDAC models, FUS combined with anti-PD-1 therapy enhanced CD8+ T cell infiltration and improved tumor control compared to monotherapy [9,15]. Similar synergy has been observed in other tumor types including melanoma and hepatocellular carcinoma when combined with PD-1, PD-L1, or cytotoxic T-lymphocyte-associated protein 4 (CTLA-4) blockade [43,60].

Beyond checkpoint inhibitors, FUS may improve delivery and immunogenicity of other modalities including oncolytic viruses, cancer vaccines, and STING agonists [61,62,63]. Ongoing trials are exploring combinations of FUS with PD-1 inhibitors, aiming to improve response rates and broaden eligibility for immunotherapy in PDAC. These early efforts support a growing rationale for integrating FUS into immunotherapy trial design as both a priming agent and adjunct.

## 8. Limitations

The clinical implementation of FUS in PDAC remains constrained by several practical and technical challenges. Anatomical positioning of the pancreas within the retroperitoneum, along with adjacent bowel, stomach, and major vessels, complicates acoustic targeting and increases the risk of unintended injury. Intervening bowel gas and rib shadowing can distort or block the acoustic pathway, making consistent energy delivery difficult. Reliable visualization and careful patient selection are therefore essential.

Tissue heterogeneity also presents a major challenge. The dense desmoplastic stroma of PDAC scatters and attenuates ultrasound energy, which can reduce the uniformity and depth of treatment, particularly with thermal ablation techniques. Mechanical approaches such as histotripsy may overcome some of these issues, but clinical data remain limited.

Technical and infrastructural barriers also persist. Real-time monitoring tools such as passive cavitation detection, MRI thermometry, and contrast-enhanced ultrasound are under development to improve treatment precision and safety. However, integration of these technologies remains limited to specialized centers. At present, there is no standardized workflow for acoustic parameter optimization across FUS platforms and substantial variability exists between devices and treatment protocols.

Clinical evidence is additionally limited by study design and regulatory complexity. Most published human studies are small, non-randomized, and conducted outside the United States. Long-term outcomes such as local control, survival, and durability of response are not well-defined, and comparative studies against other locoregional therapies have not yet been performed. Regulatory hurdles represent a major barrier to clinical implementation. Approvals are often modality- and indication-specific with separate pathways for thermal ablation, drug delivery, and immune modulation. Coordinated international efforts will be essential to establish safety benchmarks, performance metrics, and trial designs that support broader approval and clinical adoption.

Finally, important safety considerations remain incompletely characterized. Integrating FUS with current therapies introduces additional complexity, and the impact of FUS on subsequent surgical intervention remains uncertain. Tumor ablation or stromal remodeling may alter tissue planes, vascular integrity, and wound healing capacity, with unknown implications for resection feasibility, bleeding risk, and anastomotic safety. At the time of this review, there are no clinical series that report downstream operative outcomes following FUS, underscoring the need for dedicated preclinical and clinical studies evaluating surgical safety prior to broader integration in multimodal treatment pathways.

## 9. Key Knowledge Gaps

Several critical scientific questions remain unanswered and will determine the future therapeutic role of FUS in PDAC. Biomarkers capable of predicting response or guiding patient selection have not been identified. No validated imaging or circulating markers exist to quantify treatment effect, measure early response, or stratify patients by tumor biology.

The optimal integration of FUS with systemic therapies is also unknown. The sequencing, dosing, and parameters that maximize synergy with chemotherapy, checkpoint inhibitors, or sonodynamic agents have not been established. It is also unclear how different FUS modalities influence long-term stromal remodeling, tumor evolution, or immune memory in PDAC.

Finally, rigorously designed comparative trials are lacking. Whether FUS provides additive benefit over established locoregional therapies has not been tested, and the durability of the immune or stromal effects remains unknown. Addressing these knowledge gaps will be essential to determine how and when FUS should be used in multimodal PDAC care.

## 10. Future Directions

As FUS technologies mature, future research in PDAC will focus on improved patient selection, treatment personalization, and expanded clinical applications. Biomarker development is a priority. Transcriptomic and immunophenotyping may help identify tumors more likely to respond to FUS, particularly in combination with immunotherapy [16]. Circulating tumor DNA, HMGB1, calreticulin, and other DAMPs may serve as early markers of treatment response, enabling real-time adaptation during therapy [42].

Artificial intelligence and machine learning tools may emerge as powerful adjuncts for FUS treatment planning and monitoring. Algorithms can potentially optimize acoustic energy deposition by modeling tissue density, vascularity, and organ motion in real time. AI-thermal dose prediction has shown promise in other thermal ablation modalities and represents a potential future direction for FUS optimization [64].

Expanded delivery platforms such as endoscopic, laparoscopic, or robotic-assisted FUS systems may improve accessibility in anatomically complex cases. Integration with interventional radiology and endoscopy suites and operating rooms may facilitate treatment navigation and multimodal interventions.

To achieve these goals, collaborative efforts across engineering, oncology, and regulatory domains will be necessary. Investment in training, platform standardization, and multi-institutional trials will be essential to transition FUS from an investigational technology to a frontline therapeutic adjunct in PDAC management.

## 11. Conclusions

Focused ultrasound represents a versatile, non-invasive, image-guided platform with the potential to address several of the dominant biological and technical barriers that limit therapy effectiveness in pancreatic ductal adenocarcinoma. Across thermal and mechanical modalities, FUS can achieve cytoreduction while also remodeling stromal architecture, increasing vascular and cellular permeability and promoting immunogenic cell death. Preclinical evidence supports improved intratumoral delivery of chemotherapeutics and engineered agents, alongside measurable immune reprogramming that may enhance responsiveness to immunotherapy in otherwise resistant models. Early clinical experience demonstrates feasibility and acceptable safety for local tumor control and symptom palliation, with emerging approaches such as MRI-guided and EUS-guided platforms expanding access to anatomically challenging lesions.

Priorities for the field include standardized reporting of acoustic dosing and real-time feedback metrics, rigorous comparative trials that define patient-centered outcomes and survival benefit, and biomarker development to guide patient selection, treatment sequencing and response assessment. With continued refinement and integration into multimodal regimens, focused ultrasound could evolve from an investigational modality to a clinically meaningful adjunct in the management of PDAC.

## Figures and Tables

**Table 1:** Comparison of focused ultrasound modalities in PDAC. Mechanisms, clinical goals, limitations, and evidence level for thermal ablation, histotripsy, mechanical disruption, and sonoporation.

Feature	Thermal Ablation (HIFU)	Histotripsy (HIFU)	Mechanical Disruption (Pulsed HIFU)	Sonoporation (LIFU)
Mechanism	Coagulative necrosis	Non-thermal, bubble-cloud-mediated tissue fractionation	Cavitation-mediated stromal disruption	Reversible membrane permeabilization
Primary Clinical Goal	Tumor debulking, pain palliation, local tumor control	Tissue ablation, immune stimulation	Enhanced chemotherapy delivery; improved penetration through dense stroma	Enhanced chemotherapy delivery; improved drug penetration at cellular level
Limitations	Risk of thermal injury to adjacent structures	Limited acoustic access and tumor control	Risk of thermal injury to adjacent structures	Requires microbubble contrast agent; may require repeated treatments
Level of Evidence for PDAC	Multiple clinical series; no RCTs completed	Preclinical	Preclinical	Phase I/II trial completed

**Table 2:** Acoustic parameters by mechanistic modulatory of FUS in PDAC. This summary contrasts typical acoustic exposure parameters for thermal ablation, mechanical disruption, sonoporation, and histotripsy in the treatment of PDAC. Included values reflect ranges from preclinical to early clinical studies. Parameters include spatial peak pulse average intensity (ISPPA), pulse repetition frequency (PRF), and duty cycle. Values are detailed and referenced in Section 2.4 of the manuscript.

Modality	Intensity /Pressure	Frequency	Duty Cycle	Exposure Time	Key Pancreatic-Specific Parameters
Thermal Ablation	ISPPA: 1.5–2.5 kW/cm^2^; Average power: 117–399 W; Dose intensity: ≥11 kJ/cm^3^	0.96–1.5 MHz	1%	Multiple sonications over 2–4 h; Total energy: 70–1195 kJ	Requires ≥260 W average power and ≥11 kJ/cm^3^ for visible grayscale changes; intermediate intensity (2.0 kW/cm^2^) for optimal tumor response
Mechanical Disruption	Peak negative pressure: 16.5 MPa; ISPPA: 2.0 kW/cm^2^	1.5 MHz	1%	Pulse duration: 1–5 ms; Total treatment: seconds to minutes per session	Cavitation-mediated stromal disruption; enhances drug delivery 4.5 fold; minimal thermal effects
Sonoporation	Peak negative pressure: 0.05–6.55 MPa; Acoustic power: 60–200 mW/cm^2^	0.5–5 MHz	1%	Pulse duration: 4–32 μs; PRF: 10–3000 Hz; Total isonation: 0.1–900 s	Microbubble-mediated; higher power (200 mW/cm^2^) more effective; endoscopic approach achieves 6.55 MPa at 20 mm
Histotripsy	Peak negative pressure: >15 MPa (intrinsic threshold); Boiling histotripsy: shockwave-mediated	0.5–1.0 MHz	0.01–0.1%	Pulse duration: 5–30 μs; PRF: 100–500 Hz; Treatment: minutes for 1.5 cm lesion	Non-thermal mechanical fractionation; creates 1.4–1.5 cm lesions; requires clean ultrasound visualization to avoid bowel injury

**Table 3:** Clinical trials of focused-ultrasound-based approaches in pancreatic cancer.

Trial ID	Title	Modality	Patient Population	Study Design/ Status	Primary Endpoint(s)
NCT06211933	Evaluation of High-Intensity Focused Ultrasound (HIFU) Technique for Unresectable Pancreatic Tumor Treatment (PULS)	HIFU	Locally advanced, unresectable PDAC	Phase I/II, open label (active, not recruiting)	Safety/efficacy
NCT05010226	Focused Ultrasound for the Treatment of Pancreatic Cancer—an International Registry	HIFU	Pancreatic cancer (unspecified)	Not specified (not yet recruiting)	Safety/feasibility
NCT05262452	Concurrent FOLFIRINOX Plus High-Intensity Focused Ultrasound for Pancreatic Cancer	HIFU and chemotherapy	Locally advanced/borderline resectable PDAC	Exploratory, single-arm (unknown status)	Safety/efficacy
NCT04852367	PanDox: ThermoDox^®^ + Focused Ultrasound for Targeted Doxorubicin Delivery	HIFU and chemotherapy	Metastatic or locally advanced, unresectable PDAC	Phase I/II (completed)	Safety/targeted delivery effects
NCT05601323	A Study of Suizenji in Patients with Pancreatic Cancer	HIFU and chemotherapy	Metastatic or locally advanced, unresectable PDAC	Not specified (recruiting)	Safety/efficacy

## Data Availability

No new data were created or analyzed in this study. Data sharing is not applicable to this article.

## Abbreviations

The following abbreviations are used in this manuscript:

PDAC	Pancreatic ductal adenocarcinoma
FUS	Focused ultrasound
HIFU	High-intensity focused ultrasound
pHIFU	Pulsed high-intensity focused ultrasound
LIFU	Low-intensity focused ultrasound
US	Ultrasound
MRI	Magnetic resonance imaging
MRIgFUS	Magnetic resonance-guided focused ultrasound
EUS	Endoscopic ultrasound
EUS-FUS	Endoscopic ultrasound-guided focused ultrasound
CT	Computed tomography
TME	Tumor microenvironment
ICD	Immunogenic cell death
TAA	Tumor-associated antigen
DAMP	Damage-associated molecular pattern
IRE	Irreversible electroporation
RFA	Radiofrequency ablation
MWA	Microwave ablation
SBRT	Stereotactic body radiation therapy
GEMM	Genetically engineered mouse model
ISPPA	Spatial peak pulse average intensity
MPa	Megapascal
ROS	Reactive oxygen species
PD-1	Programmed cell death protein-1
PD-L1	Programmed death-ligand 1
CTLA-4	Cytotoxic T-lymphocyte-associated protein 4
